# Incidence of community onset MRSA in Australia: least reported where it is Most prevalent

**DOI:** 10.1186/s13756-019-0485-7

**Published:** 2019-02-12

**Authors:** Jessica K. Cameron, Lisa Hall, Steven Y. C. Tong, David L. Paterson, Kate Halton

**Affiliations:** 1 Australian Centre for Health Services Innovation and the Institute for Health and Biomedical Innovation, Queensland University Technology, Brisbane, Australia; 2 School of Public Health, University of Queensland, Brisbane, Australia; 3 Victorian Infectious Disease Service, The Royal Melbourne Hospital, and Doherty Department University of Melbourne, Peter Doherty Institute for Infection and Immunity, Victoria, Australia; 4 Menzies School of Health Research, Darwin, Australia; 5 UQ Centre for Clinical Research, University of Queensland, Brisbane, Australia

**Keywords:** *Staphylococcus aureus*, Methicillin resistant *Staphylococcus aureus*, Community, Antimicrobial resistance, Australia

## Abstract

**Background:**

This is the first review of literature and synthesis of data on community onset methicillin resistant *Staphylococcus aureus* (CO-MRSA) infections in Australia. Incidence of CO-MRSA varies considerably in Australia, depending on geographic and demographic factors.

**Methods:**

Data for the rates of MRSA infections were collected from articles identified using PubMed, Scopus, the grey literature and data from State and Federal Government Surveillance Systems. We synthesized data and developed a framework for how data was selected, collated, linked, organized and interpreted.

**Results:**

The results of our literature search demonstrates considerable gaps in the reporting of CO-MRSA in Australia. Consequently, total incidences were under reported; however the available data suggests the incidence varied between 44 (Tasmania) and 388 (southern Northern Territory) cases per 100,000 person years. Hospitalised cases of CO-MRSA varied between 3.8 (regional Victoria) and 329 (southern Northern Territory). Taking the median percentage of infections by site for all regions available, skin and soft tissue infections (SSTIs) consisted of 56% of hospitalized CO-MRSA, compared with bacteremias, which represented 14%. No region had a complete data set of CO-MRSA infections treated in out-patient settings and so incidences were underestimates. Nevertheless, estimates of the incidence of CO-MRSA treated outside hospitals varied between 11.3 (Melbourne) and 285 (Northern Territory) per 100,000 person-years. These infections were chiefly SSTIs, although urinary tract infections were also noted.

Incidences of CO-MRSA blood-stream infections and outpatient skin and soft tissue infections have been increasing with time, except in Tasmania. CO-MRSA is observed to affect people living in remote areas and areas of socioeconomic disadvantage disproportionately.

**Conclusions:**

We generated the first estimates of the incidence of CO-MRSA infections in Australia and identified stark regional differences in the nature and frequency of infections. Critically, we demonstrate that there has been a lack of consistency in reporting CO-MRSA and a general dearth of data. The only government in Australia that requires reporting of CO-MRSA is the Tasmanian, where the infection was least prevalent. Some regions of Australia have very high incidences of CO-MRSA. To improve surveillance and inform effective interventions, we recommend a standardized national reporting system in Australia that reports infections at a range of infection sites, has broad geographic coverage and consistent use of terminology. We have identified limitations in the available data that hinder understanding the prevalence of CO-MRSA.

**Electronic supplementary material:**

The online version of this article (10.1186/s13756-019-0485-7) contains supplementary material, which is available to authorized users.

## Background

*Staphylococcus aureus* is carried asymptomatically by half (range 29–84%) [1–4] the population and frequently causes minor skin infections.

Compared with methicillin-susceptible *S. aureus* infections, methicillin-resistant *S. aureus* (MRSA) infections have been associated with increased hospitalisation rates, [5] increased mortality [6, 7] and greater delays in receiving active antimicrobial therapy, [5, 8] resulting in poorer outcomes, including increased mortality and increased length of stay. [9–12]

Historically, MRSA has been associated with healthcare associated infections (HAI); however, broad-scale infection prevention efforts have reduced hospital onset (HO) HAI infections and the circulation of strains traditionally associated with healthcare. [13, 14] More recent evidence suggests most MRSA infections are now arising in the community. [13, 15] Rates of MRSA infections appear to be increasing [14, 16, 17] faster than rates of growth of population [18] or healthcare utilisation. [14]

Community onset (CO-MRSA) infections are defined as cases either identified in a primary healthcare setting or are cases where symptoms were observed and a pathology sample positive for MRSA was collected within 48 h of hospitalisation. Compared to HAI MRSA, CO-MRSA infections have been associated with increased risk of metastatic seeding; decreased chance of empirical antimicrobials being effective and increased duration of antibacterial therapy. [19] CO-MRSA bloodstream infections (BSIs) are more likely to require admission to an intensive care unit. [19] Patients with CO-MRSA infections tend to be younger and often are otherwise healthy. [19, 20]

The incidence of CO-MRSA infection across Australia has not been documented, but it is known to vary geographically. [7] Only in Tasmania is reporting of CO-MRSA mandatory. The Australian Group on Antimicrobial Resistance has regularly reported on the number of MRSA BSI cases from 32 institutions from each state and territory, with the most recent report specifying number of community onset cases. [21] Previous nation-wide studies have reported the incidence of *S. aureus* bacteremia (SAB), estimating that in Australia, 61 to 77% of SABs were CO, 72% of MRSA SABs were CO and 13% of all SABs were CO-MRSA. [21, 22]

Hence, incidences of CO-MRSA infections cannot be calculated from currently available routinely reported data. International Classification of Diseases (ICD-10) diagnosis data has been found to be unreliable for such specific diagnoses, lacking sensitivity and having low positive predictive value for measuring the number of hospital acquired infections. [23–25] The Australian Institute of Health and Welfare (AIHW) recently released a report on SABs, but omitted location of onset and susceptibility to methicillin. [26]

We here generate the first estimate of the national incidence of CO-MRSA infections, by synthesizing data collated from academic literature and government reports. We identify regional differences in the nature and frequency of infection. We recommend changes in measurement and reporting to enable future assessment of key questions.

## Methods

### Literature search

**Fig. 1:**
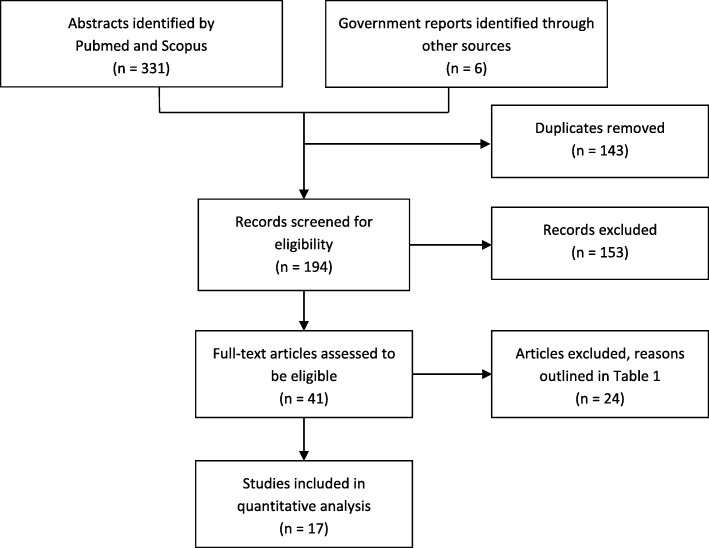
Flow chart detailing the study screening and selection process

The screening and selection process of the literature review is outlined in Fig. 1 and described in the Additional file 1.

We sought studies that published numbers of all infections in a defined region or population and time. We searched the academic literature between January 2000 and April 2016, using PubMed and Scopus and the terms “community” and “onset”, “acquired” or “associated”, combined with “methicillin resistant *Staphylococcus aureus*” and “Australia”. Furthermore, we sought literature from State and Federal Government Health Department websites and consulted authors and specialists for grey literature.

We excluded clinical case studies and studies that excluded adults, did not include data from Australia, began before 2000, did not have a defined period of data collection, required consent from individual patients, or did not provide information on whether the infection onset occurred in hospital or in the community. If more than one data set was available for a region, we included those that excluded infections with healthcare associated risk factors, as defined by Kallen et al. [27]; or if two data sets used the same definitions for the same region, the most recent was included.

“Site” is used to refer to the anatomical site of an infection. “Region” or “area” refers to a geographic space to which a data set relates. “Location” refers to where the patient was at the time of onset of the infection.

### Study populations

**Fig. 2:**
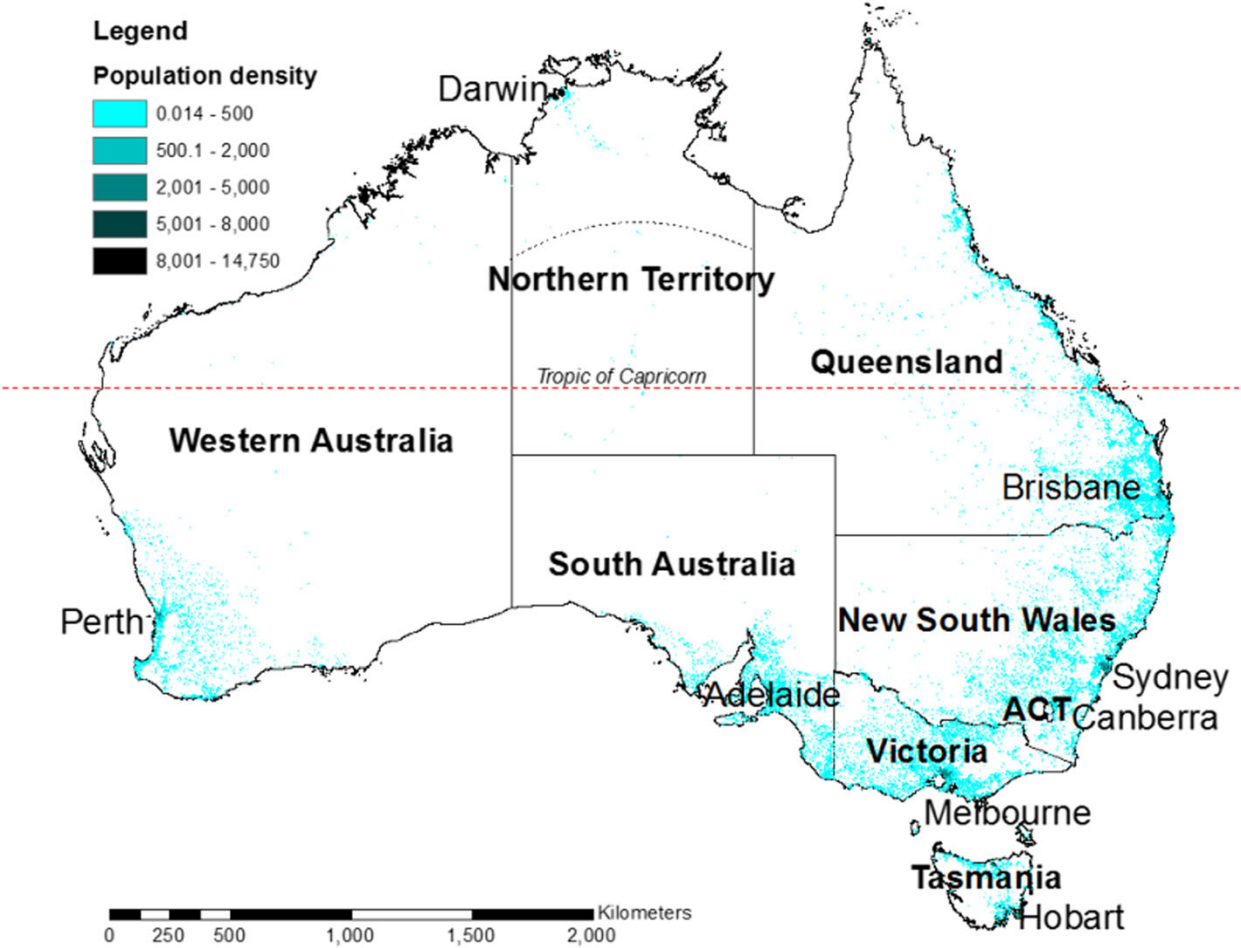
Map of Australia showing the population density (from ABS); states and territories, capital cities and the Tropic of Capricorn

The data were analyzed for each state or territory. Northern Territory (NT) data arose from two sub regions: The Top End and the Alice Springs region, as shown by the dotted line in Fig. 2 and defined using the Statistical Local Areas from the Australian Bureau of Statistics (ABS) that best reflect the local health districts.

### Data synthesis

Number of infections, denominator population and duration of data collection were required to estimate incidence. Where available, the specified catchment population was used as denominator population. Otherwise, the catchment population or area was sought from state health departments or hospital websites and ABS census data for that area and time period was used.

Incidence of BSIs, lower respiratory tract (LRTI) and skin and soft tissue (SSTI) infections were calculated, generally using numbers of infections by site. Total incidences were calculated using total number of patients with an infection. Incidence for infections listed as “other”, musculoskeletal, bone or joint, endocarditis or “of other sterile cavities” were not calculated because of lack of data, clarity or consistency of definitions between publications; however, these infections were included in the totals. The incidence of infections at these sites were expected to be considerably lower than that of SSTIs.

Incidence was calculated separately for cases treated as inpatients (IPs), outpatients (OPs) and at emergency departments (EDs). Where necessary, it was assumed that all BSIs and LRTIs were admitted, based on expert advice (personal communication: M. O’Sullivan, January 12, 2016; S. Tong, November 11, 2015 and G. Coombs, October 1, 2015).

## Results

### Literature search

All studies included in this review specified that the data represented clinically significant isolates and excluded screening swabs, duplicates and repeat cultures from the same patient within 14 days. In two publications, [13, 28] discharge diagnoses for some cases were indicative of a HAIand these cases were excluded.

CO infections were generally defined as an infection detected within 48 h of admission to hospital, using hospital records. Many studies also excluded patients with various healthcare associated risk factors from the definition of CO or indicated the number of cases of HAIs included as CO. [19, 20] Some publications excluded specific cases as noted in the footnotes to Table 1, below.

**Table 1:** Publications on CO-MRSA infections in Australia used to calculate incidences in this study

Citation	Region^a^	Data collection period	Admission status	Site of infection^b^	Data source(s)^c^	Additional exclusions^d^	Publication type^e^
Nimmo 2013 [13]	Queensland	2011	IP,OP,ED	All	P,D	T	PRJ
Stevens 2006 [28]	Alice Springs, NT	2005–6	IP,OP,ED	All	R,D	T	PRJ
Agostino 2016 [20]	Hunter - New England, NSW	2008–14	IP,OP,ED	Total	R	T	Th
Mitchell 2009 [46]	Tasmania	2008–9	IP,OP,ED	Total	S	s,c,p,d,t	GR
Tong 2009 [15]	Top End, NT	2006–7	IP,ED	Total	P	t,Other	PRJ
Wehrhahn 2010 [8]	Perth – Fremantle, WA	2005–7	IP	All	P	s,c,p,d,o,t	PRJ
Bennett 2007 [34]	Small hospitals Victoria	2004–5	IP	Total	S	T	PRJ
Marquess 2013 [35]	Queensland	2005–2010	IP	BSI	R	NS	PRJ
Robinson 2009 [19]	Perth, WA	1997–2007	IP	BSI	R	t,Other	PRJ
Laupland 2013 [33]	ACT	2000–8	IP	BSI	NS	NS	PRJ
Strachan 2014 [18]	Victoria	2009–13	IP	BSI	S	–	GR
Wells 2014 [32]	Tasmania	2008–14	IP	BSI	S	s,c,p,d,t	GR
Tong 2015 [17]	Northern Territory	2008–12	OP	SSTI	C	NS	PRJ
Bennett 2014 [36]	Melbourne, Vic	2006	OP	SSTI	C	L,t	PRJ
Pandey 2008 [39]	Bowral, NSW	2004	ED	SSTI	NS	NS	LE
Lim 2014 [40]	The Alfred Hospital, Vic	2003–11	ED	BSI	NS	m,t	PRJ

^a^Australian Capital Territory (ACT), Victoria (Vic)

^b^All: data for SSTIs, LRTIs, BSIs and others listed separately, Total: only data for total infections were provided

^c^R: retrospective review of laboratory database, P: prospectively identified cases for inclusion, C: Community pathology database, S: surveillance system, D: discharge diagnosis, NS: not stated

^d^ s: surgical procedure – some authors required that the infection was at the surgical site c: therapy for cancer (variously: receiving IV chemotherapy, being an oncology patient, neutropenia or immunosuppressive medication), p: percutaneous or indwelling device, d: dialysis, t: time since previous discharge (variously ≤48 h to ≤12 months), L: residence in long term care facility, m: children (either less than 18 or 20 years old), o: other, NS: none stated. Further exclusion criteria included history of *S. aureus* infection, being an intravenous drug user, organ transplant recipients, respiratory and burns patients, receiving plasmapheresis, home IV therapy or employment in healthcare

^e^ *PRJ* peer-reviewed academic article, *Th* thesis, *GR* government report, *LE* letter to the editor

Few data were available on CO-MRSA by site of infection, as shown in Table 1, often because site-specific data included nosocomial cases. Criteria for diagnoses were seldom provided; however, one study identified pneumonia using radiographic evidence in addition to a positive culture of respiratory fluids or blood. [8] Generally, BSIs were identified by positive blood culture, with some studies requiring symptoms. [29] SSTIs were usually identified by type of pathology specimen. [14, 28] There was a wealth of data on BSIs, two studies specifically on LRTIs [29, 30] and one on infective endocarditis [31].

### Data synthesis – Incidence calculations

Data used to estimate incidence was either: incidence of CO-MRSA, [18, 32, 33] numbers of CO-MRSA infections in a given period of time, [20, 34–36] numbers of MRSA infections and proportions that were nosocomial or CO, with and without HAI risk factors or, finally, the number of CO *S. aureus* infections and the proportion of those that were MRSA.

### Inpatient populations

Total incidences and incidences for hospitalized CO-MRSA infections are presented in Table 2, with percentages of the populations who are Indigenous, who live in remote areas and who live in areas with an average Index of Relative Socioeconomic Disadvantage (IRSD) in the lowest decile in Australia, indicating socioeconomic disadvantage.

**Table 2:** Incidence of hospitalized (IP) CO-MRSA infections by site of infection and for various regions, calculated using published numbers of infections over defined time periods

Region	Population size used in calculations	Data collection period	Incidence (/100000 person-years)					References	Demographic factors		
			Total	Total IP	IP SSTI	LRTI	BSI (1^°^ and 2^°^)		% Indigenous [47]	% Living in remote areas [48]	% Living in area with lowest IRSD decile [49]
Top End	176,000 [15]	2006–7		81				[15]	26	28	20
Queensland	4,500,000 [13]	2005–11	95.5	26	22.8	2.8	3.7	[13, 35]	4	3	9
Alice Springs	51,000 [28]	2005–6,14	388	329	184	31	20	[28, 50]	43	100	33
Hunter - New England	752,952 [20, 51]	2008–14	146					[20, 52]	5	0.3	11
Perth – Fremantle	470,389 [53, 54]	1997–2007		6.1	2.1	1.1	4.2	[8, 19]	2	0.02	2
ACT	370,000 [33]	2000–8					2	[33]	2	0	1
Regional Victoria	617,692 [55]	2004–5		3.8				[18, 46]	2	0.4	11
Victoria	NS	2009–13					> 1.6	[18]	1	0.1	9
Tasmania	489,958 [55, 56]	2008–14	44				1	[32, 46]	4	2	17

Table 2; Total incidence and incidence of hospitalized (IP) CO-MRSA infections by site of infection and for various regions, calculated using published numbers of infections over defined time periods.

Availability of data was highly regional, with ample data from the central and northern regions, a paucity of data from the most populous states of Victoria and New South Wales (NSW) and no publications from South Australia or regional Western Australia (WA), which neighbor regions of high incidence.

Data for BSIs was available for most state or territory capitals and three states, representing over half the population of Australia. Aside from BSIs, approximately a quarter of the population were represented by some form of data: either total CO-MRSA infections, total inpatient CO-MRSA infections or one of SSTIs or LRTIs.

BSIs that were secondary to SSTIs or LRTIs were included in Table 2 as both sites of infection, but only once in the total column. Many BSIs occurred simultaneously with infections of other sites not included in this analysis, such as bone and joint infections. Together, SSTIs, LRTIs and BSIs accounted for nearly all patients.

Incidence varied greatly by region. The Alice Springs region had the highest rates, with an incidence of inpatient SSTIs four times that of the Top End. The Top End had second highest rates, with incidences over three-fold that of its neighbor, Queensland. The southern-most regions of the ACT, Victoria and Tasmania experienced low incidences of CO-MRSA BSIs. The proportions of infection at each anatomical site in Queensland were similar to the proportions in Alice Springs.

Consistent with previous studies a higher incidence of CO-MRSA was observed for locations with a higher proportion of people identifying as Indigenous, those with more socio-economic disadvantage, and those living in more remote locations [15, 37, 38].

### Outpatient populations

The incidence of CO-MRSA infections that were not hospitalized is shown in Table 3. The sources of data on infections treated as outpatients were variously from private community pathology services, state coordinated pathology laboratories or a hospital pathology service.

**Table 3:** Incidence of CO-MRSA infections treated as outpatients not presenting to an emergency department, by site of infection and for various regions, calculated using published numbers of infections over defined time periods

Region	Population size used in calculations	Data collection period	Data source	Incidence (/100000 person-years)			References
				Total	SSTI	UTI	
Northern Territory	211,945 [51]	2008–12	Community	285			[17]
Queensland	4,500,000 [13]	2011	State		64.5		[13]
Alice Springs	51,000 [28]	2005–6	Hospital	145	141	4	[28]
Melbourne	1,796,296 [56]	2006	Community	11.3	10.8		[36]

Although limited, the outpatient data suggests similar geographical trends to those observed in inpatients; specifically: high incidence in NT and Alice Springs, moderate in Queensland and relatively low in Melbourne and Tasmania. Most outpatient CO-MRSA cases detected were SSTIs.

### Emergency department

All presentations of MRSA to ED were considered CO. Only three papers offered data on presentations of MRSA to emergency departments. In regional NSW in 2004, the incidence of CO-MRSA in skin abscesses presenting to ED was 80 / 100,000 person-years. [39] At The Alfred Hospital in Melbourne between 2003 and 2011, the average incidence was 0.6 / 100,000 person-years. [40] The incidence of CO-MRSA presenting to ED in the Top End was 29 / 100,000 person-years in 2006–7. [15] This estimate required the assumption that all nosocomial cases and colonizations detected were included in the percentage reported as hospitalizations. It was also assumed that those not hospitalized had presented to ED.

### Pathways to seek care

Methicillin resistance is only identifiable by pathology services and so all data used were from or linked to pathology databases. The multiplicity of care-seeking behaviors resulted in some patients’ infections being detected by a pathology service that did not reflect their admission status or being registered by more than one pathology laboratory. Some data coming from hospital pathology laboratories are from patients presenting to and receiving treatment in primary care. Similarly, some patients whose infections were identified by community pathology services were admitted.

Assuming all BSIs are hospitalized, slightly less than half of all CO-MRSA BSIs in Melbourne initially presented to ED, based on the incidences above.

### Incidence over time

**Fig. 3:**
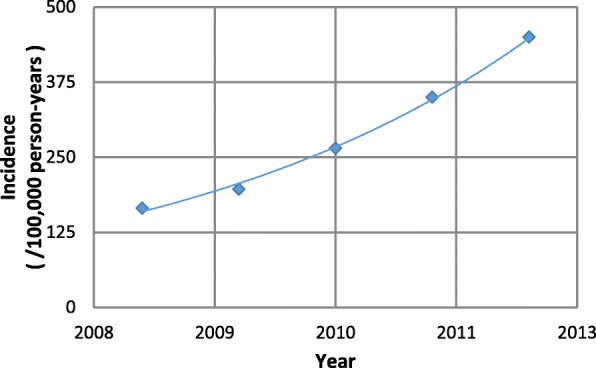
Incidence of CO-MRSA SSTIs collected by a community pathology service provider in NT (personal communication, S. Tong January 20, 2016). [17]

**Fig. 4:**
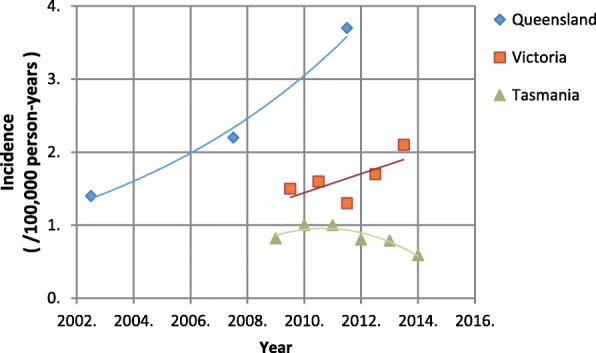
Incidence of CO-MRSA BSIs in Queensland, Victoria and Tasmania. [13, 14, 18, 32, 35]

Some incidence data were available for multiple time points. A study by Tong and colleagues [17] gave outpatient incidences at multiple time points in NT (S. Tong, personal communication, January 20, 2016), as shown in Fig. 3. Figure 4 presents the incidence at different time points of BSIs in Queensland, [13, 14, 35] Victoria, [18] and Tasmania [32]. Additionally, the incidence of all CO-MRSA infections in Tasmania increased from 39 in 2008 to 49 /100000 person-years in 2009. Except for BSIs in Tasmania, there appears to be an increasing trend with time in the incidence of CO-MRSA infections, both in inpatient and outpatient cases.

## Discussion

Incidence of CO-MRSA in parts of Australia is comparatively high. In 2011, a cross-section of localities in the United States had adjusted incidences of CO-MRSA of 5.3 (total) and 4.8 (inpatient) per 100,000 person-years. [41] The definitions of CO and HAI used in the US study more closely resembled that of the Perth-Fremantle study, where rates were lower. However, in the US study, the total incidence of all CO, with and without healthcare associated risk factors, both crude and adjusted was comparable to Queensland, which excluded patients with the key HAI risk factors, and was much lower than the NT rates. Laupland and colleagues [33] found that in the period 2005–2008, various regions in Scandinavia and Canada had adjusted incidences of CO-MRSA BSIs of, respectively, less than 0.3 and between 1.8 and 5.4 infections per 100,000 person-years, while the ACT had an adjusted incidence of 2 per 100,000 person-years, which was amongst Australia’s lowest.

### Data synthesis

Geographical differences are consistent with previous reports of MRSA, both HO and generally. [14, 16] The higher rates have been associated with ethnicity, remoteness and socio-economic status. [15, 37, 38]

Health system structure and availability and care-seeking behaviors may explain differences in incidence between regions. Alice Springs experienced considerably higher inpatient incidence than the Top End, possibly because access to outpatient care was limited for patients who lived remotely in southern NT. This may also account for the considerably higher incidence of CO-MRSA SSTIs presenting to ED in rural NSW compared with the incidence of all CO-MRSA presenting to ED in the Top End.

Most CO-MRSA infections were not admitted to hospital. In Queensland, the outpatient data were incomplete, nevertheless the outpatient incidence was 2.5 times that of inpatient incidence. Uniquely, a publication from Tasmania captured all CO-MRSA infections in the state - but did not distinguish whether cases were admitted or not. If, however, we assume that the ratio of BSIs to total inpatient infections in Tasmania was the same as in Queensland, then the incidence of outpatient CO-MRSA was 4.5-fold that of inpatient CO-MRSA.

Essentially all CO-MRSA treated as outpatients were SSTIs and a significant majority of admitted cases were SSTIs, except in Perth. This suggests that surveillance of CO-MRSA SSTIs is warranted. The incidence of LRTIs relative to BSIs varied by location, perhaps because different criteria were used to identify infections or difficulties in collecting pathology samples.

CO is now defined by the Centers for Disease Control as infections that occur within 72 h of admission; however, only one of the studies met this definition.

### Limitations

There was a paucity of data in the academic literature, particularly on key indicators of morbidity such as site of infection and admission status. Lack of specificity in reporting CO infections has resulted in the inclusion of community onset infections both with and without healthcare associated risk factors in the data used to calculate incidences. Most publications lacked a breakdown of the number of infections by anatomical site. Given the difficulty of obtaining pathology specimens from patients with LRTIs, the number of cases of CO-MRSA LRTIs was probably a significant underestimate. For SSTIs, data were available for the absolute number of MRSA isolations. However, the denominator number of tests was typically not reported or available and hence it is not clear the degree to which changes in the incidence of isolation of MRSA is related to changes in testing practices.

### Data sources

Most studies used data from pathology departments of public hospitals; however, a key indicator of a patient’s morbidity is whether they were ultimately admitted to hospital, [8, 39, 40, 42] which is not included in most pathology data sets. Therefore, to determine final admission status, pathology data needs to be linked to hospital records. Alternatively, assumptions can be used to estimate the proportion of cases of SSTIs and urinary tract infections (UTIs) that were hospitalized. In Queensland, patients’ admission status at the time a positive pathology sample was collected was used as a proxy for whether an SSTI case was hospitalized, thus overestimating the proportion of outpatients and underestimating inpatients.

As the community clinic system is multifarious and multiple private pathology services exist, no study captured all and only cases that were treated in the community. The multiple pathology services mean that, except in Tasmania, the ED and outpatient data reported were incomplete and underestimated the burden, leaving most infections unrepresented by the available data.

### Recommendations

To understand the true burden of CO-MRSA, a national reporting system is required. Surveillance ought to be geographically comprehensive, since populations with highest prevalence are often the most difficult to survey, being remote and small in size. [43] Cases treated in primary care settings need to be captured as a large proportion of infections are not admitted to hospital. Currently available data focusses on BSIs; data collection needs to take in different infection sites and types to fully recognize the burden of disease. Finally, all information should be shared and available for analysis. In many states, HO or HAI MRSA is notifiable, suggesting that CO cases presenting at hospitals must be vetted for location of onset. This vetting process produces valuable information on CO infections that is not utilized currently.

Consistent terminology would also be advantageous. “Community-associated strains” continues to be used, although these strains are no longer associated with community acquisition [6, 14, 15, 44] and international recognition that it is inaccurate terminology. [45] The terms “multiresistant” and “non-multiresistant MRSA strains” are widely understood and highlight the significance of these strains.

## Conclusions

We identified a paucity of data, a lack of consistency of definitions and highly regionalized data collection, resulting in an absence of information on the prevalence of CO-MRSA in populations where it is expected to be highest. Data on the most prevalent type of infection, SSTIs, were sparse, particularly in outpatient settings.

Reported incidence of CO-MRSA was high compared to other, demographically similar countries. Consistent, nationwide reporting of CO-MRSA cases is necessary to understand its true incidence and plan control strategies.

We recommend requirements for reporting MRSA, including the reporting of a wider range of infection sites, better geographic coverage and consistent use of terminology.

## Additional file

Additional file 1: Further information on the literature identified and exclusions. (DOCX 21 kb)

## Abbreviations

ABS: Australian Bureau of Statistics
AIHW: Australian Institute of Health and Welfare
BSI: Blood stream infection
CO: Community-onset
CO-MRSA: Community onset Methicillin Resistant *Staphylococcus aureus*
ED: Emergency department
GP: General practice
HAI: Healthcare associated infection
HO: Hospital onset
ICD-10: International Classification of Diseases
IP: Inpatients
IRSD: Index of Relative Socioeconomic Disadvantage
LRTI: Lower respiratory tract infection
MRSA: Methicillin Resistant *Staphylococcus aureus*
NSW: New South Wales
NT: Northern Territory
OP: Outpatient
*S. aureus*: *Staphylococcus aureus*
SAB: *S. aureus* bacteremia
SSTI: Skin and soft tissue infection
UTI: Urinary tract infection
WA: Western Australia
