# Supplementary material for: Incidence of community onset MRSA in Australia: least reported where it is Most prevalent
Source: Antimicrob Resist Infect Control. 2019 Feb 12;8:33. doi: 10.1186/s13756-019-0485-7 (PMC6373119; doi:10.1186/s13756-019-0485-7)
Supplement: Supplementary file 1 — Further information on the literature identified and exclusions. (DOCX 21 kb) [file 13756_2019_485_MOESM1_ESM.docx]

# Supplementary material

Searches of the academic literature produced 188 unique publications. An initial review found that only 41 of these included relevant data, collected since January 2000. A detailed review of the publications excluded a further 24 publications, as shown in [Table](#Ref4487573011) S.1. Five reports from state government departments (S1, S2, S3) and one thesis chapter (S4) were also found; however, two of the reports were excluded. Properties of the final publications included in the data synthesis are presented in [Table](#Ref4484127992) 1, [in](#Ref4484139641) the main text.

Table S.1; Number of publications excluded and the reference of each, stratified by reason for exclusion.

| **Number excluded** | **Reason** | **References** |
| --- | --- | --- |
| 6 | Numbers of MRSA and CO-SA, but not CO-MRSA | (S5, S6, S7, S8, S9, S10) |
| 6 | More recent data were available | (S11, S12, S13, S14, S15, S16) |
| 3 | Length of the period of data collection was not clear | (S17-S19) |
| 3 | Pediatric focus | (S20-S22) |
| 2 | Subpopulation with specific comorbidity | (S23, S24) |
| 1 | Required consent | (S25) |
| 1 | Alternative publication with better fitting definitions available | (S26) |
| 1 | Another publication available with longer data collection period | (S27) |
| 1 | Denominator population not available | (S28) |

## References for Supplementary Material

1. Mitchell, B., et al., Tasmanian acute public hospitals healthcare associated infection surveillance report. 2009, Department of Health and Human Services: Hobart, Tasmania.

2. Strachan, J. and M. Easton, Staphylococcus aureus bloodstream and cerebrospinal fluid infections in Victoria, 2009-2013, in Victorian Infectious Diseases Bulletin. 2014: Melbourne, Vic. p. 68-72.

3. Wells, A., et al., Tasmanian acute public hospitals healthcare associated infection surveillance report. 2014, Department of Health and Human Services: Hobart, Tasmania.

4. Agostino, J., The changing epidemiology of methicillin resistant Staphylococcus aureus infection in north-eastern New South Wales and implications for management. 2016, Hunter New England Health.

5. Munckhof, W.J., et al., Methicillin-susceptible, non-multiresistant methicillin-resistant and multiresistant methicillin-resistant Staphylococcus aureus infections: a clinical, epidemiological and microbiological comparative study. Eur J Clin Microbiol Infect Dis, 2008. 27(5): p. 355-64.

6. Nimmo, G.R. and G.W. Coombs, Community-associated methicillin-resistant Staphylococcus aureus (MRSA) in Australia. Int J Antimicrob Agents, 2008. 31(5): p. 401-10.

7. Tong, S.Y., et al., Impact of ethnicity and socio-economic status on Staphylococcus aureus bacteremia incidence and mortality: a heavy burden in Indigenous Australians. BMC Infect Dis, 2012. 12: p. 249.

8. Turnidge, J.D., et al., Staphylococcus aureus bacteraemia: a major cause of mortality in Australia and New Zealand. Med J Aust, 2009. 191(7): p. 368-73.

9. Turnidge, J.D., et al., Epidemiology and outcomes for Staphylococcus aureus bacteraemia in Australian hospitals, 2005-06: report from the Australian Group on Antimicrobial Resistance. Commun Dis Intell Q Rep, 2007. 31(4): p. 398-403.

10. Boan, P., et al., Epidemiological, clinical, outcome and antibiotic susceptibility differences between PVL positive and PVL negative Staphylococcus aureus infections in Western Australia: a case control study. BMC Infect Dis, 2015. 15(1): p. 10.

11. Nimmo, G.R., et al., Changing epidemiology of meticillin-resistant S. aureus in Queensland, Australia, 2000-2006: use of passive surveillance of susceptibility phenotypes. J Hosp Infect, 2008. 70(4): p. 305-13.

12. Aung, A.K., et al., Changing epidemiology of bloodstream infection pathogens over time in adult non-specialty patients at an Australian tertiary hospital. Commun Dis Intell Q Rep, 2012. 36(4): p. E333-41.

13. Peleg, A.Y., et al., Life-threatening community-acquired methicillin-resistant Staphylococcus aureus infection in Australia. Eur J Clin Microbiol Infect Dis, 2005. 24(6): p. 384-7.

14. Thomas, R., et al., Community-acquired methicillin-resistant Staphylococcus aureus pneumonia: a clinical audit. Respirology, 2011. 16(6): p. 926-31.

15. Douglas, M.W., et al., Epidemiology of community-acquired and nosocomial bloodstream infections in tropical Australia: a 12-month prospective study. Trop Med Int Health, 2004. 9(7): p. 795-804.

16. Gosbell, I.B., et al., Non-multiresistant and multiresistant methicillin-resistant Staphylococcus aureus in community-acquired infections. Med J Aust, 2001. 174(12): p. 627-30.

17. Coombs, G.W., et al., Community-onset Staphylococcus aureus Surveillance Programme annual report, 2012. Commun Dis Intell Q Rep, 2014. 38(1): p. E59-69.

18. Coombs, G.W., et al., Prevalence of MRSA strains among Staphylococcus aureus isolated from outpatients, 2006. Commun Dis Intell Q Rep, 2009. 33(1): p. 10-20.

19. Howden, B.P., et al., Community and health-care associated non-multiresistant methicillin-resistant Staphylococcus aureus in Victoria. Med J Aust, 2005. 183(10): p. 548.

20. Britton, P.N. and D.N. Andresen, Paediatric community-associated Staphylococcus aureus: a retrospective cohort study. J Paediatr Child Health, 2013. 49(9): p. 754-9.

21. Engelman, D., et al., Invasive Staphylococcus aureus Infections in Children in Tropical Northern Australia. Journal of the Pediatric Infectious Diseases Society, 2014. 3(4): p. 304-311.

22. Roediger, J.C., et al., Paediatric Staphylococcus aureus bacteraemia: A single-centre retrospective cohort. Journal of Paediatrics and Child Health, 2016.

23. Hayashi, Y., et al., Influenza-associated bacterial pathogens in patients with 2009 influenza A (H1N1) infection: impact of community-associated methicillin-resistant Staphylococcus aureus in Queensland, Australia. Intern Med J, 2012. 42(7): p. 755-60.

24. Sy, R.W. and L. Kritharides, Health care exposure and age in infective endocarditis: results of a contemporary population-based profile of 1536 patients in Australia. Eur Heart J, 2010. 31(15): p. 1890-7.

25. Rémond, M.G.W., et al., Community-acquired pneumonia in the central desert and north-western tropics of Australia. Internal Medicine Journal, 2010. 40(1): p. 37-44.

26. Hewagama, S., T. Spelman, and L.J. Einsiedel, Staphylococcus aureus bacteraemia at Alice Springs Hospital, Central Australia, 2003–2006. Internal Medicine Journal, 2012. 42(5): p. 505-512.

27. Harch, S.A.J., et al., High burden of complicated skin and soft tissue infections in the Indigenous population of Central Australia due to dominant Panton Valentine leucocidin clones ST93-MRSA and CC121-MSSA. BMC Infect Dis, 2017. 17(1): p. 405.

28. Coombs, G.W., et al., Australian Staphylococcus aureus Sepsis Outcome Programme annual report, 2013. Commun Dis Intell Q Rep, 2014. 38(4): p. E309-19
